# The use of EMDR in positive verbal material: results from a patient study

**DOI:** 10.3402/ejpt.v7.30119

**Published:** 2016-07-04

**Authors:** Suzy Johanna Martina Adriana Matthijssen, Marcel van den Hout

**Affiliations:** 1 Altrecht Academic Anxiety Centre, Utrecht, The Netherlands; 2 Clinical Psychology, Utrecht University, Utrecht, The Netherlands; 3 Dutch Institute of Forensic Psychiatry and Psychology, Utrecht, The Netherlands

**Keywords:** EMDR, working memory theory, positive verbal material, verbal imagery, positive closure, modality-specific taxing, eye movements

## Abstract

**Background:**

According to the working memory (WM) theory of eye movement desensitisation and reprocessing (EMDR), dual tasks that tax WM during memory recall reduce image vividness and emotionality of memory during future recalls when no dual task is carried out. There is some evidence that WM taxing also reduces vividness and emotionality of auditory or verbal imagery.

**Objective:**

The present study tests the effect of eye movements (EM) on positive verbal material (verbal imagery), which is used in different parts of the EMDR protocol. In the Dutch version of the standard EMDR protocol, a procedure “Positive Closure” (PC) is performed, which uses verbal imagery under dual task condition (EM). The value of EM in this procedure has not been established and according to the WM account would be counterproductive. Two earlier studies with undergraduates, with a set-up comparable to the present one, showed no additive value of the EM in the procedure, but no counterproductive effect either.

**Method:**

Thirty-six patients rated the belief in possessing two positive personality traits and emotionality of the traits. They then had an EMDR session targeting a negative memory and recalled and re-rated the belief and emotionality of the traits afterward. Subsequently, they recalled one trait while dual tasking (EM) and the other trait without dual tasking. Afterward, they re-rated the belief and emotionality.

**Results:**

EM did not affect the belief in possessing the trait or the emotionality. Secondary analysis shows an effective EMDR session itself enhances the belief in the traits, compared to a less or non-effective EMDR session.

**Conclusions:**

EM are not effective in enhancing the belief in possessing a personality trait or the emotionality. If replicated by other patient studies, this suggests elimination of the PC procedure.

**Highlights of the article:**

- In this study the additive value of EM on verbal material (in the procedure Positive Closure) is not found.
- No effect of the procedure Positive Closure on the Belief in a personality trait nor the emotional intensity of the trait was found.
- An effective EMDR session (large reduction of the SUD during the session) was effective in changing the Belief in personality traits.

Since the introduction of eye movement desensitisation and reprocessing (EMDR) in 1989 (Shapiro, 1989), the field has moved a long way from scepticism toward this therapy to viewing it as an evidence-based intervention for posttraumatic stress disorder (PTSD; see Chen et al. (2014) for a recent meta-analysis) proven equally effective as trauma-focused cognitive behavioural therapy (Bisson et al., 2007). A study by De Jongh, Ernst, Marques, and Hornsveld (2013) even suggests that it is effective in resolving negative memories that play a role in, or underlie, a broad variety of psychological symptoms and conditions. The core feature of EMDR therapy is that the patient is asked to hold a disturbing image of a negative memory in mind while engaging in sets of eye movements (EM) or other bilateral stimuli, such as taps or tones (Lee & Cuijpers, 2013; Shapiro, 2001). The additive value of the EM – in comparison to recall only – was established in numerous studies, and a recent meta-analysis of Lee and Cuijpers (2013) showed the effect size for the additive effect of EM in EMDR treatment studies to be significantly moderate (Cohens *d*=0.41) and significantly large in laboratory studies (*d*=0.74).

Still there is a lot of debate about the working mechanism of EMDR and why EM are effective. In the original description of EMDR (Shapiro, 2001) it was assumed that the bilaterality, which was induced by the horizontal EM (or bilateral tones or taps), was a necessity to ensure effective treatment. However, evidence is accumulating that supports an explanation based on working memory theory (WMT). The theory predicts that any dual task that taxes working memory during memory recall will reduce the vividness and emotional intensity of mental images. Two tasks (keeping the image in mind and the other taxing task) compete for the limited working memory capacity (Baddeley, 2012). Moving one's eyes from side to side while recalling a memory would, according to the WMT, leave less capacity for the memory. As a result, the memory would become less vivid and emotional, and the image is reconsolidated as such (van den Hout & Engelhard, 2012). The WMT implies that the crucial part of EMDR would be that the traumatic experience is reprocessed while a *distracting* stimulus is given, not necessarily a *bilateral* stimulus. This is confirmed by studies showing efficacy of vertical EM (Gunter & Bodner, 2008), drawing a complex figure (Gunter & Bodner, 2008), playing the computer game Tetris (Engelhard, Van Uijen, & van den Hout, 2010), mental arithmetic (Engelhard, van den Hout, & Smeets, 2011; van den Hout et al., 2010), calculating out loud (Kemps & Tiggemann, 2007), and mindful breathing (van den Hout et al., 2011). It is stressed that this is not the only explanation for the working mechanism of EMDR and for the relief of trauma in general. There are many explanations given for the mechanism behind EMDR itself and for the relief of symptoms. Also, TF-CBT is an evidence-based therapy for the relief of trauma symptoms. Both TF-CBT and EMDR have the “exposure” element, but a distinct feature is EM which in EMDR have proven their additive value (Lee & Cuijpers, 2013).

EMDR typically targets negative visual imagery, but also seems to affect vividness and emotionality of positive visual imagery. A study of van den Hout, Muris, Salemink, and Kindt (2001) showed that, compared to control conditions that did not (or hardly) tax working memory, positive memories were rated less positive by 60 undergraduates after EM. A study of Barrowcliff, Gray, Freeman, and MacCulloch (2004) showed engagement in EM compared to the eyes stationary (ES) condition resulted in significant reductions on measures of vividness and emotional valence for both positive and negative autobiographical memories in 80 participants (20 community participants and 60 undergraduates).

In a study of Engelhard et al. (2010) 60 undergraduates recalled negative and positive memories in three conditions: recall only, recall with EM, and recall with playing Tetris. Before and after these conditions, vividness, emotionality, and physiological startle responses during recall were measured. For positive memories, EM and Tetris decreased startle responses compared to recall only. In addition, EM decreased emotionality but Tetris did not, and Tetris decreased vividness but EM did not.

Hornsveld et al. (2011) also reflected on positive visual imagery. They evaluated the effects of EM on positive memories such as those used in the Resource Development and Installation (RDI) protocol. The RDI protocol is an EMDR-related procedure developed to strengthen positive associations in positive and resourceful memories (Korn & Leeds, 2002). Fifty-three university undergraduates were asked to recall three positive memories (memories representing pride, perseverance, and self-confidence, respectively) under three conditions: horizontal EM, vertical EM, and a control condition. Vividness, emotionality, and subjective strength of the resource were measured. Both types of EM reduced the vividness, emotionality, and also the subjectively experienced strength of the positive memories, indicating that EM were counterproductive (Hornsveld et al., 2011).

However, a contradictory result was found by Keller, Stevens, Lui, Murray, and Yaggie (2014). They studied the effect of EM on positive personal memories and their results indicated an increase in memory strength and vividness. Different in their design, ratings were conducted after 1-minute processing periods, whereas other studies did not include such periods.

In summary, most research indicates that both negative and positive visual imagery are rated less vivid and emotional after recall+EM. But does this also hold true for *auditory* or *verbal* imagery? To the authors’ knowledge two studies assessed auditory images. Baddeley and Andrade (2000) conducted seven studies of which five included auditory images. The auditory images consisted of novel sequences of tones, familiar sounds or bizarre sounds. All of the studies used healthy participants and they rated the auditory images on vividness. In all five studies, participants were asked to hold the auditory stimulus in mind as an image under dual task conditions and then to rate its vividness. Dual task conditions were either auditory or visual suppression or a control condition. Auditory images were rated less vivid after dual task suppression. An interaction between modality of imagery and concurrent task occurred, with the rated vividness of auditory images being reduced to a greater extent by the auditory suppression than by the visual suppression. A limitation of the study is that Baddeley and Andrade used only vividness ratings and did not include emotionality ratings, and the imagery used did not have meaningful autobiographical content.

A study by Kemps and Tiggemann (2007) also included auditory images. The authors conducted a study in which 68 undergraduates were instructed to specifically form visual or auditory images and were asked to rate the vividness and emotionality of the images. The memory was recalled three times in succession, each time in a different dual task condition (a control condition, EM, and articulatory suppression). Auditory images were rated less vivid and emotional after dual task suppression and concurrent modality-specific taxation (articulatory suppression for auditory images and EM for visual images) reduced vividness and emotional intensity ratings in both auditory and visual images to a greater extent.

Positive auditory material (verbal imagery) is used at different moments of the standard EMDR protocol, for example, installing the future template and installing the positive cognition. Installing the future template is a procedure where a feared non-harmful situation is being visualised while pronouncing the sentence “I can handle this” and at the same time EM are performed, until the point where the patient feels capable to face the feared situation in real life (e.g., walking in the street where a person once was robbed). Installing the positive cognition is the procedure where, after desensitising a negative image a positive cognition (e.g., “I can handle this”, “I am safe”, and “I am a good person”) is pronounced while still visualising the prior selected negative image and simultaneously performing EM. In the Dutch version of the standard EMDR protocol another procedure that addresses verbal material called “Positive Closure” is added at the end of the session, to enhance belief or faith in possessing relevant personality traits (Matthijssen & van den Hout, 2016). This is an adaptation to the original EMDR protocol and may deviate from other international versions of the EMDR protocol. The procedure addresses solely verbal imagery under dual task condition (EM). The procedure is not considered a core part of the EMDR procedure, but is added to strengthen a patient's belief in self-statements with respect to the progress made in the EMDR session. In their first study, Matthijssen and van den Hout (2016) compared the belief in possessing two selected personality traits in 30 undergraduates under two different conditions: EM and a control condition (ES). After exposure to an EMDR session the participants were asked to select two positive personality traits and to rate the *belief* in the chosen traits. While recalling the traits participants were exposed to EM or ES, the order of which was counterbalanced. A second study, with a sample population of 46 undergraduates also addressed the same procedure where two personality traits were recalled under two conditions (EM or ES), but the intervention was not precipitated by an EMDR session, to rule out any positive bias toward EM. In this study besides *Belief* in the trait, *Emotionality* was added as a dependent variable to test if the selected material was emotional. Results in both studies showed that, regardless of the condition, there was no significant difference between pre- and post-test measurements, neither for Belief nor for Emotionality. The utility of EM in the Positive Closure procedure was not supported by these laboratory findings. A limitation of both studies is that undergraduates in a non-clinical setting were studied. Possibly, the positive closure procedure *is* effective under clinical conditions, that is, during real EMDR. Certainly, the latter is the assumption that underlies the procedure. However, note that extrapolating from the findings discussed above on the negative effects of EM on positive visual imagery, one might expect negative effects of EM on positive closure as well.

Thus, the aim of the present study was to test whether, in PTSD patients who were eligible for trauma-focused psychotherapy with EMDR, positive closure+EM affects the belief in possessing a personality trait. To obtain sufficient statistical power (0.8, with a confidence interval of 95% and an expected medium effect size, *f=*0.25), 34 patients were needed. This would also address whether the procedure PC is of any value in the EMDR protocol. A second aim was to assess whether the course of the EMDR session (within session improvement) influences the belief in (and emotionality of) the positive trait.

## Method

### Patients

Data from 36 patients were collected. They had a mean age of 39.1 years (*SD=*11.4), with an age range from 19 to 60. For more patient characteristics see Table 1. Inclusion criteria were being diagnosed with PTSD and being eligible for trauma-focused psychotherapy with EMDR. No patients were excluded from the study and there were no drop-outs. Since therapists asked their patients to participate, the researchers do not know if and how many patients refused participation.

**Table 1:** Patient characteristics (*N*=36)

Age		(*M* = 39.1, *SD* = 11.4)
Gender		
Female		72.2% (*N* = 26)
Male		27.8% (*N* = 10)
Comorbid disorders		
Comorbid Axis I disorder	Depression	52.8% (*N* = 19)
	Bipolar disorder	8.3% (*N* = 3)
	Panic disorder	5.6% (*N* = 2)
	Cognitive disorder	2.8% (*N* = 1)
	Vaginism	2.8% (*N* = 1)
	Undifferentiated somatoform disorder	2.8% (*N* = 1)
Comorbid Axis II disorder	Personality disorder NOS	22.2% (*N* = 8)
	Avoidant personality disorder	5.6% (*N* = 2)
	Borderline personality disorder	2.8% (*N* = 1)
Education level		
Primary school		2.8% (*N* = 1)
Secondary school		33.3% (*N* = 12)
Lower vocational education		2.8% (*N* = 1)
Middle vocational education		22.2% (*N* = 8)
Higher vocational education		16.7% (*N* = 6)
University		19.4% (*N* = 7)
Missing		2.8% (*N* = 1)
Psychopharmacological drugs		
Antidepressants		47.2% (*N* = 17)
Antipsychotics		19.4% (*N* = 7)
Anti-epileptics		8.3% (*N* = 3)
Hypnotics		5.6% (*N* = 2)
Anti-histaminic medication		5.6% (*N* = 2)

No selection was made in the type of trauma of the patients (which encompassed sexual violence, physical violence, physical accidents, war trauma, physical violence in childhood, sexual violence in childhood, neglect, and other trauma), length or quantity of the trauma. The years since index trauma varied from 9 months up to 35 years. The number of prior EMDR sessions varied from it being the first session up to 30 sessions (*M*=6.8, *SD*=7.4). Twenty patients were treated at the Altrecht Academic Anxiety Centre, where complex anxiety disorders are treated. At the centre patients are diagnosed through a thorough assessment procedure, where they are subjected to The Structural Clinical Interview for DSM-IV for axis I (SCID-I and on request The Structural Clinical Interview for DSM-IV for axis II [SCID-II]). The other patients that were treated mostly in smaller settings were diagnosed by their therapists who were all experienced EMDR therapists and are expected to have the skills to diagnose their patients either with an axis I or axis II diagnosis.

### Design

The study had a 2 (Time: T2 and T3) by 2 (Condition: eye movements (EM) and eyes stationary (ES)) repeated measures within-subject design. Dependent variables were “Belief in possessing the positive relevant personality trait” (Belief) and “Emotional intensity” (Emotionality). All patients selected two relevant personality traits from the Personality Characteristics List (see Materials) and while thinking through one or the other trait, they were exposed to two conditions: eye movements (Recall+EM) and eyes stationary (Recall only), the order of which was counterbalanced. There was also a counterbalance for strength of the Belief, resulting in four conditions (see Procedure). The study was conducted by certified EMDR therapists. A third measurement moment (T1 at the beginning of the EMDR session) was included for answering the second question about EMDR sessions affecting the belief in possessing the trait. A one-way ANOVA was conducted with Time (T1 and T2) as the independent variable.

### Materials

#### List of personality characteristics

For the purpose of the study, patients selected two positive personality traits. A list of relevant personality traits was created based on the Dutch version of the standardised short version of the BIG Five (Gerris et al., 1998). The trait “emotionally stable” was replaced by “energetic” and “positive attitude.” Patients were also given the opportunity to mention a relevant personality trait that was not on the list. This list is the same list used in earlier studies of Matthijssen and van den Hout (2016).

#### Visual analogue scales

The use of the 10-cm visual analogue scales (VAS) was also replicated from the earlier studies by Matthijssen and van den Hout (2016). To measure belief in possessing the relevant personality trait the patients were asked “To what extent do you believe that you possess this personality trait right now?” and to put a mark on a 10-cm VAS ranging from 0 (not believing) to 10 (completely believing) at three specific time moments (T1, T2, T3). Emotionality was also measured on a 10 cm VAS ranging from “not pleasant at all” (0) to “very pleasant” (10) by asking the question: “If you think about how much the trait is applicable to you, how pleasant is that for you?” The numerical values 0 and 10 were visible on the actual scales. At T2 and T3 ratings, participants were not able to see their previous scoring.

#### SUD-difference scores

Subjective Units of Disturbance (SUD) is a scale that measures the subjective intensity of disturbance or distress felt by the individual at that moment of time, from 0 (none at all) to 10 (maximum distress). This was verbally rated by the patient. The effectiveness of the desensitisation phase (“EMDR effectiveness”) was measured by SUD-difference scores which were calculated by measuring the SUD of the image before start of the desensitisation phase and measuring the SUD after the desensitisation phase and subtracting the last from the first score.

#### EMDR protocol

For the study, the Dutch version of the standard EMDR protocol was used (Ten Broeke & De Jongh, 2012). The EMDR protocol consists of eight steps: (1) introduction, (2) assessment, (3) desensitisation, (4) installation, (5) body scan, (6) future template, (7) Positive Closure, and (8) reassessment (of the session). The study focusses on the seventh step: Positive Closure. The standard procedure involves the question “What is the most positive or valuable thing you have learned about yourself during this last hour/this last session, with regard to this theme or this event?” The patient mentions a personality trait, and this is then re-formulated into an “I-statement” (e.g., “I am strong”). Once the statement is formulated, a set of 20–25 left–right–left eye-movements are performed by the patient. After the set the patient is asked if any other positive things arise or spring to mind. If yes, then another 20–25 left–right–left eye-movements are offered up until the point when the patient doesn't mention any new positive qualities or relevant personality traits. A modification was made in this standard procedure to allow for two conditions per patient. The patient was asked at the start of the session – and not once arriving at the seventh step – “What are the *two* most valuable things that *you want to learn* about yourself during this next hour/this session, with regard to this theme or this event?” The patient was asked to evaluate both traits on Belief and Emotionality. Then the normal EMDR session was executed and once arrived at the seventh step in the protocol the patient was asked to take the two selected traits back in mind and re-evaluate the traits on Belief and Emotionality. After exposing the patients to the conditions (EM and ES) the question to re-evaluate the traits on Belief and Emotionality was posed one more time.

#### Attention checklist

A checklist was given after the last re-evaluation of the traits on Belief and Emotionality. In the checklist patients were asked how much they held the trait in mind while being exposed to one of the two conditions. This variable was measured on a 10 cm VAS with poles from 0 to 10.

### Procedure

*Ethical statement*: The study was conducted by Dutch EMDR therapists on their own patients and the sessions were performed in the therapist's room. Most of the patients received treatment at the Altrecht Academic Anxiety Centre in Utrecht, the Netherlands and the research was approved by the committee of scientific research of Altrecht (a mental health institution). Other approached EMDR therapists (from the Dutch association of EMDR) conducted the research mostly in smaller (private) practices. There were 11 therapists from the Altrecht Academic Anxiety Centre involved and 8 therapists from outside Altrecht, of which 7 saw patients in their private practice. One therapist saw patients in a large mental health institution. The research was conducted according to the principles expressed in the Declaration of Helsinki. There were no invasive techniques used or substance administration given. In giving consent, patients indicated to have read and to have agreed with both the rules regarding participation and the researchers’ commitments and privacy policy. They were also informed that they could stop participating at any time, without consequences.

Patients were given an oral and written briefing that provided information about the research. This was done by the therapist of the patient in the session before executing the study or at the start of the session itself. When patients decided to participate they signed an informed consent. The EMDR session was carried out as usual, with the standard EMDR protocol with the minor alterations mentioned. When the session time was over – regardless in which step of the protocol the patient was – or when the therapist arrived at step 7, the slightly altered procedure “Positive Closure” was given. The patient was asked to re-evaluate the two selected traits for both Belief and Emotionality. After this the therapist differentiated between the most and the least believed in trait in order to counterbalance for strength and order. The trait with the highest/lowest score, and the order of the two conditions (EM and ES), was counterbalanced. The patients were informed which trait the experiment would commence with, and the trait was then stated out loud. They recalled the trait while performing EM simultaneously or while performing the control condition. In the EM condition, participants were exposed to 20–25 horizontal left–right–left EM which were evoked by following the top of the researcher's fingers. Therapists were instructed to move the fingers as fast as possible as long as the patient could still follow the fingers of the therapist. The control task was to look at the top of the researcher's fingers for a duration of 15 seconds, which was approximately the same interval as the EM condition. After the exposure to one of the two conditions the participants were asked to re-rate to the Belief and Emotionality. The procedure was then repeated for the other condition. Finally the patient had to evaluate how much they thought about the traits during the intervention (EM or ES). After this they were given a debriefing form with more details and background about the research itself, and also the contact possibilities with the researcher responsible for the study. After this the session was either closed or another image was selected to desensitize.

## Results

### Effects of positive closure with or without EM on the Belief and Emotionality

For the main research question – to test the effect of EM on the belief in possessing a positive personality trait – data was analysed with a 2 by 2 repeated measures ANOVA. The independent variables were Time (T2 and T3) and Condition (EM and ES). The dependent variable was *Belief in possessing the personality trait* (Belief). There was no significant main effect of Time (*F*(1, 35)=1.167, *p*=0.287) or Condition (*F*(1, 35)=0.071, *p*=0.792) and no significant interaction effect between Time× Condition (*F*(1, 35)=0.109, *p*=0.743) was found. Thus, the belief in possessing the trait was not affected by the procedure, neither with, nor without EM. Also a 2 by 2 ANOVA was executed for Emotionality. No main effect for Time (*F*(1, 35)=0.575, *p*=0.453), Condition (*F*(1, 35)=0.017, *p*=0.897) or an interaction effect (*F*(1, 35)=0.610, *p*=0.440) was found. Emotionality of the positive trait did not change from T2 to T3, regardless of the condition (EM or ES). The results can be found in Table 2.

**Table 2:** Mean and standard deviation of the VAS-scores on the variables Belief and Emotionality at T1, T2, and T3 for both conditions (EM and ES)

	EM		ES	
		
	Belief (*SD*)	Emotionality (*SD*)	Belief (*SD*)	Emotionality (*SD*)
T1	4.29 (2.95)	6.23 (3.05)	4.08 (2.98)	5.87 (3.50)
T2	4.94 (2.91)	6.27 (2.99)	4.87 (3.37)	6.43 (3.07)
T3	5.19 (3.12)	6.64 (2.89)	5.04 (3.25)	6.40 (2.97)

### Effects of the EMDR session on Belief and Emotionality

Note that from T1 to T2 the groups were treated equally. Therefore, the scores on the two traits were averaged per person. A one-way ANOVA with Time (T1, T2) as an independent variable and Belief as a dependent variable showed an effect for Time (*F*(1, 35)=4.792, *p*=0.035), suggesting that Belief differed from T1 to T2; Table 2 shows that Belief went up from T1 to T2. Also for *Emotionality* a one-way ANOVA was conducted, but no significant effect (*F*(1,35)=0.630, *p*=0.433) was found there, showing that *emotional intensity* of the personality trait did not differ between T1 and T2.

**Fig. 1:**
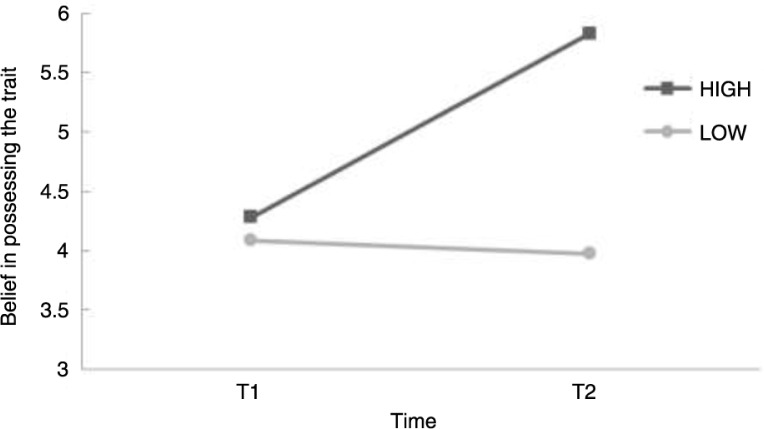
To visualise the effect of the SUD scores a median split was conducted. LOW and HIGH SUD-difference scores are displayed. Scores are the Belief from T1 to T2.

Possibly, the increased belief in possessing the trait was related to the efficacy of the EMDR session that was conducted between T1 and T2. There was a strong negative correlation between the decrease in SUD and the increase in the Belief (*r*=−0.494, *p*<0.002), indicating that the degree of within session improvement (decrease in negativity of the trauma image) was accompanied by an increase in the belief in the positive characteristic between T1 and T2. This however did not affect the Emotionality from T1 to T2. To test to what degree the difference in Belief was explained by the SUD-difference score, the latter was added as a covariate to the ANOVA. There was an effect of the SUD-difference score (*F*(1,35)=10.998, *p*=0.002), but the main effect of Time disappeared after entering the covariate (*F*(1,35)=1.248, *p* = 0.272) which means the increase in Belief scores from T1 to T2 can be explained by the decrease in SUD scores. Figure 1 visualises the effect of the SUD scores on belief. A median split was conducted to visualise this. LOW SUD-difference scores represent scores <−4,25 and HIGH SUD-difference scores represent scores >–4,25. Another interesting question would be if the procedure PC would be more helpful for patients who ended the session with a high SUD. The procedure could possibly be more helpful for those who have an unfinished session. The difference in belief scores between T2 and T3 when adding “SUD end scores” as a covariate were analysed. Adding the “SUD end scores” showed no significance (*F*(1,35)=0.640, *p*=0.803) in change of Belief scores from T2 to T3.

### Attention to the trait during the condition

**Fig. 2:**
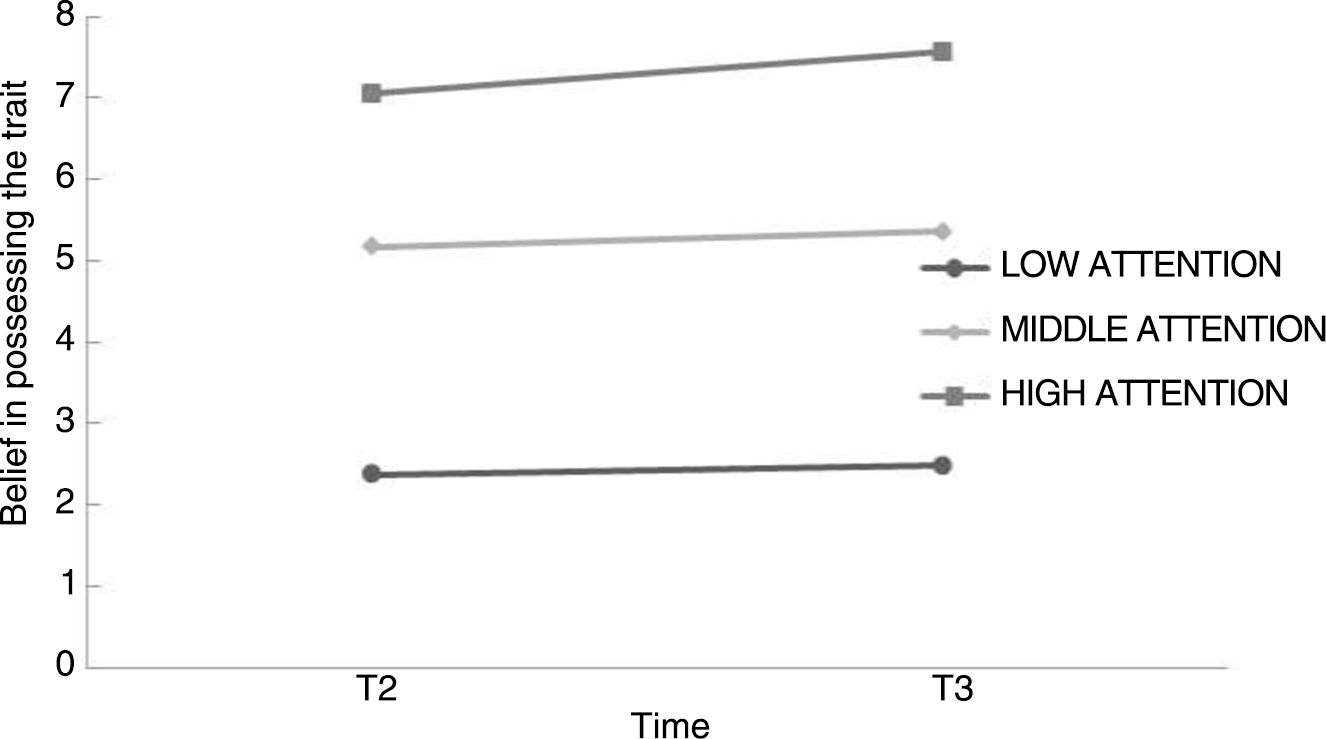
To visualise the effect of paying attention three groups were created (LOW, MIDDLE, and HIGH ATTENTION). Scores are the Belief from T2 to T3.

Participants were classified as low, middle, or high attenders based on their scores on the VAS (*M=*6.37, *SD=*3.08). Low attention represent scores from 0 to 3.29 (>–1 *SD*). Middle attention is 3.29–9.44 (between –1 *SD* and +1 *SD*) and high attention represent scores from 9.44 to 10 (>+1 *SD*). Examining whether paying attention to the trait while performing the dual task (EM) was influencing Belief was tested by conducting a mixed factorial 3 (between factor; low attention, middle attention, high attention) ×2 (within factor; T2, T3) design with Belief as the dependent variable. No interaction-effect was found for Time × “attention to the trait” (*F*(1,35)=0.169, *p*=0.845). Thus the pattern reported earlier, no effects of positive closure with or without EM, remained unchanged when the attention paid to the trait during the procedure was taken into account (see Fig. 2).

## Discussion

The aim of the study was to test whether EM had an effect on the strength of positive verbal material as used in the procedure Positive Closure (PC). Two positive personality traits were selected and recalled under two conditions: EM (standard in PC) and a control condition, ES. According to the WMT one might assume EM would produce a decrease in belief in possessing the personality trait (Belief) and emotional intensity (Emotionality), but inferred from earlier research (Matthijssen & van den Hout, 2016) no effect of EM may be predicted. The results of the present study are in line with earlier research. No effect of EM during PC on the belief in possessing the positive personality trait, or emotionality was found. EM appears to be ineffective in the procedure, and also the control condition shows no effect on Belief and Emotionality. Note that the present findings replicate two earlier analogue experiments but that the present study had a very high clinical/ecological validity. We studied PTSD patients treated with real life EMDR administered by trained and skilled EMDR therapists. If replicated by other patient studies, we suggest eliminating the PC procedure from the protocol at least in the way as it is now performed. It does not relate to elimination of the installation of the positive cognition, as this is a different procedure being performed and was not examined in this study.

What does seem to have an effect on the belief in possessing the trait is the success of the prior EMDR session. Patients with a strong decrease in SUD scores during the desensitization phase had an increase in belief in possessing the trait during the session. When SUD scores decrease during the session one can assume that the patient feels better, relieved or less disturbed. This in turn could lead to a better feeling about oneself and an increase in the belief in possessing the positive personality trait. This does not explain why Emotionality ratings do not change during the EMDR session. It appears then that during EMDR sessions, the idea of having certain positive traits remains as pleasant, but what changes is the belief in actually having such positive traits.

In summary, positive closure does not have any additional effect on beliefs and emotionality of positive self-statements and the belief in a positive self-statement is correlated with the decrease of distress due to the EMDR procedure.

Of course, the reported belief in the positive trait may reflect a state effect and the increase in belief may disappear once the emotional state is replaced by other ones. Alternatively, the increased belief may, to some extent, persist and it would be worthwhile to document whether a series of EMDR sessions are attended by between-session increases in believability of positive traits. No effects were found of paying attention to the trait during PC, with means that there was no difference in Belief scores between patients who paid a lot of attention and patients that did not or hardly paid attention.

Why no effect is found on “emotional intensity” even from T1 to T2 remains unclear. Maybe the question “If you think about how much the trait is applicable to you, how pleasant is that for you?” was difficult for patients to answer.

Some limitations of the current study may be noted. First the order of the EMDR protocol was slightly altered. The positive cognitions were asked before the actual EMDR session was started; this differs from the usual protocol where the cognitions are elicited after the sixth step of the EMDR protocol. Meanwhile there is no *a priori* reason to suspect that this alteration affected the results achieved. A second alteration was that a list of positive personal characteristics was given to the patients, wherefrom they were asked to choose two positive characteristics, where in the usual EMDR session the patient is not presented a list of characteristics. One may argue that asking the patient to mention a desired personality trait (e.g., “resilient”) may be sub-optimal and that asking to formulate some current state (e.g., “I am doing well”) may be more helpful. But again, and certainly given the robustness of the null-effects observed here and earlier, there is no reason to assume that this alteration affected the results observed. Another limitation of the study was that no information was collected on participation rates, so no information was collected about how many and what kind of patients refused participation. This generates a potential source of bias for patients included. A final limitation concerns confirmation of PTSS diagnoses of patients included. Patients who were included from private practices were diagnosed by the therapists in the practices, but were not assessed by structured clinical interviews to confirm diagnoses. However, the therapists were experienced trauma therapists and insurers expect them to diagnose patients.

An explanation for the lack of effect from EM could be attributable to the type of memory that is used. The present relevant personality traits do not relate to any single episode and are, by their very nature, generic. Furthermore, it is an abstract idea and not a concrete picture. It remains unclear if or to what extent making EM during the recall of generic information and abstract ideas reduces the actual vividness or emotionality of that information during future recalls. Future research should address this question.

Furthermore, modality-specific taxing – meaning taxing in the same modality (verbal material verbally and visual material visually) – was not used. No modality-specific taxing was added because the researchers wanted to stay as close as possible to the original “Positive Closure” procedure. Earlier studies (Baddeley & Andrade, 2000; Kemps & Tiggeman, 2007) showed a (small) beneficial effect of modality specific taxing. Here the taxing was conducted in the opposite modality – a visual taxation on an auditory image. This may partly explain the absence of the effects of EM.

Moreover, the lack of results could be that the manipulation was not strong enough. Only 20–25 left–right–left EM were conducted, as this was the standard PC-procedure. Perhaps multiple sets of EM should be performed to test the effect of EM on positive verbal material. Future research could easily tackle this.

Finally, note that the occurrence of null-results may always be attributed to some potential artefact. The present findings were collected under ecologically valid conditions. They show that the positive closure procedure as routinely carried out has no detrimental but also no beneficial effect, which questions adding the procedure in its current form. Of course one might study under what conditions the positive closure procedure (with or without EM) may still prove beneficial. Given the robustness of the present findings, such studies may not be a promising endeavour.
